# Deep imaging in the brainstem reveals functional heterogeneity in V2a neurons controlling locomotion

**DOI:** 10.1126/sciadv.abc6309

**Published:** 2020-12-04

**Authors:** Joanna Schwenkgrub, Evan R. Harrell, Brice Bathellier, Julien Bouvier

**Affiliations:** 1 Université Paris-Saclay, CNRS, Institut des Neurosciences Paris-Saclay, 91190 Gif-sur-Yvette, France.; 2 Institut Pasteur, INSERM, Institut de l’Audition, 63 rue de Charenton, F-75012 Paris, France.

## Abstract

V2a neurons are a genetically defined cell class that forms a major excitatory descending pathway from the brainstem reticular formation to the spinal cord. Their activation has been linked to the termination of locomotor activity based on broad optogenetic manipulations. However, because of the difficulties involved in accessing brainstem structures for in vivo cell type–specific recordings, V2a neuron function has never been directly observed during natural behaviors. Here, we imaged the activity of V2a neurons using micro-endoscopy in freely moving mice. We find that as many as half of the V2a neurons are excited at locomotion arrest and with low reliability. Other V2a neurons are inhibited at locomotor arrests and/or activated during other behaviors such as locomotion initiation or stationary grooming. Our results establish that V2a neurons not only drive stops as suggested by bulk optogenetics but also are stratified into subpopulations that likely contribute to diverse motor patterns.

## Teaser

Calcium imaging of genetically defined neurons in the ventral brainstem during locomotion reveals unexpected functional diversity.

## INTRODUCTION

Locomotion is an essential behavior in most animals, which allows them to confront external obstacles and pursue goals. While the spinal cord circuits that elaborate the coordinated movements of the limbs required for locomotion have been extensively studied (*1*–*4*), the descending pathways that orchestrate their activation are still poorly characterized. Reticulospinal (RS) neurons of the brainstem reticular formation (RF) with direct projections to the spinal cord are key candidates for controlling the initiation, maintenance, and arrest of locomotion (*5*–*10*). Yet, the functional diversity of RS neurons in locomotor control has been hard to precisely define because of the heterogeneous composition of the RF and its ventral location, altogether making cell type–specific recordings a daunting task with conventional approaches.

Functional neuronal subtypes that are intermingled in the brainstem, including in the RF, can be accessed using developmentally expressed transcription factors (*11*–*18*). V2a neurons, defined by Chx10 expression (*18*, *19*), are a genetically circumscribed neuronal class present throughout the ventral spinal cord and the brainstem. In the former, they represent locally projecting, candidate pre-motoneurons, involved in the control of left-right coordination of limb movements (*20*, *21*). In the brainstem, V2a neurons populate all antero-posterior levels of the medullary and pontine RFs and might have diverse functions and projection profiles. Initially linked to breathing control through local projections in the RF (*22*), they also represent a subset of excitatory RS neurons that collectively innervate multiple spinal segments, in both mice and zebrafish (*15*, *16*, *23*). In mice, broad optogenetic activation of V2a neurons in the gigantocellular reticular nucleus (Gi) causes an arrest of ongoing locomotion, while broad silencing favors mobility (*15*). These effects were attributed to V2a neurons with direct projections to the hindlimb controllers in the lumbar spinal cord. This cell class, termed V2a “stop neurons,” may thus control the episodic nature of locomotion.

However, to date, there are still no direct observations of the endogenous activity of V2a stop neurons during natural behaviors, and while bulk optogenetic perturbations are informative, they can only deliver a crude understanding of functionality. If synchronous optogenetic activation of all V2a Gi neurons causes locomotor arrests, this does not imply that all V2a neurons are synchronously and systematically activated at naturally occurring locomotor arrests. Despite this limitation, broad optogenetic activations of V2a Gi neurons were first delivered by a single optical fiber positioned at the midline, to aim for bilateral activations (*15*). However, in very recent work, unilaterally restricted activations of V2a neurons located medially were shown to steer locomotor trajectory (*24*). Whether these distinct functional outcomes reflect the bilateral (former study) or unilateral (latter study) activation of the same cells or instead a functional segregation of medially and laterally located V2a neurons in controlling locomotion remains to be investigated. Last, some V2a brainstem neurons may also control locomotor initiation (*16*, *23*) and/or other motor actions (*22*). Therefore, this neuronal class may, similarly to other transcription factor–defined classes (*25*), contain distinct functional subtypes. Monitoring activity profiles of individual cells during behavior can help resolve functional diversity within a spatially confined neuronal ensemble (*26*). In principle, this can be achieved by combining genetically targeted expression of fluorescent calcium indicators with functional imaging at cellular resolution, but its application to the brainstem RF during locomotion has not been achieved.

Here, we established deep and single-cell resolution imaging of V2a Gi neurons in the RF of freely moving mice using a head-mounted miniature microscope (*26*–*29*). We found that locomotor arrest was correlated with increased activity in up to half of V2a Gi neurons. However, activations were unreliable in single neurons, suggesting that individual locomotor stops rely on a fraction of V2a neurons. Functional diversity was also present, as some neurons were inhibited at locomotor arrest and/or activated in other behaviors such as locomotor initiation or stationary grooming. Our results indicate that V2a neurons are a heterogeneous and dynamic population that likely orchestrates multiple motor programs.

## RESULTS

### Imaging the activity of V2a Gi neurons

**Fig. 1:**
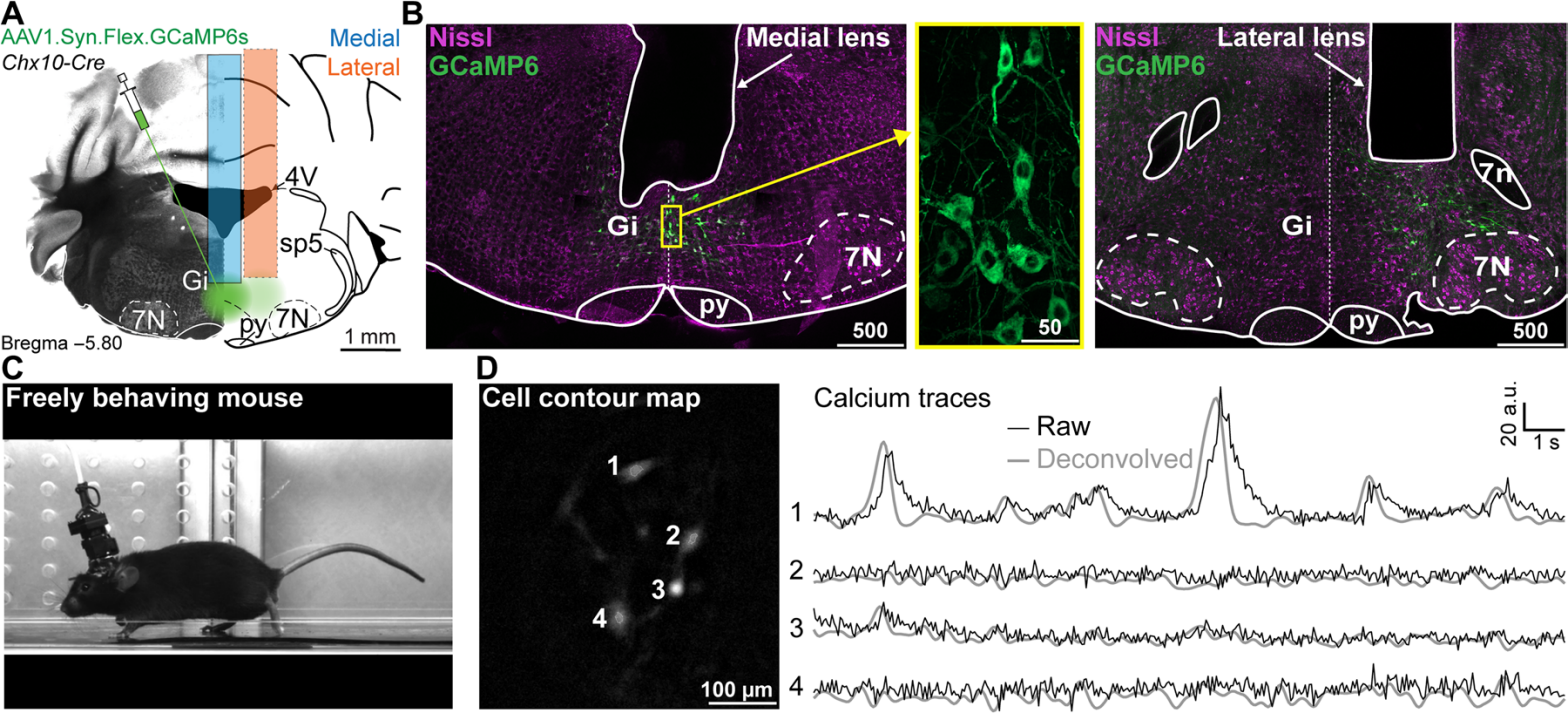
Activity of V2a neurons can be recorded with micro-endoscopy. (**A**) Schematic of viral injections and lens implantation into the Gi of a *Chx10-Cre* animal for calcium imaging in freely behaving mice. (**B**) Nissl-stained (magenta) coronal sections of *Chx10-Cre* mouse showing native GCaMP6s fluorescence (green) and the location of medial (left) or lateral (right) GRIN lens implants. Inset in the yellow box is a magnified view of GCaMP6s expression in V2a neurons. Scale bars in micrometers. (**C**) Example mouse during treadmill running with miniature microscope attached. Photo credit: Joanna Schwenkgrub, CNRS. (**D**) Example cell contour map (left) with corresponding raw/deconvolved calcium traces (right). Abbreviations used in (A) and (B): 4V, 4th ventricle; 7N, facial motor nucleus; 7n, facial nerve; py, pyramidal tract; Gi, gigantocellular reticular nucleus; sp5, spinal trigeminal tract; a.u., arbitrary units.

All experiments were performed on a validated transgenic mouse line in which the expression of Cre recombinase is restricted to V2a neurons [*Chx10-Cre*; see (*15*, *30*, *31*)]. To monitor the endogenous activity of individual V2a neurons during behavior, we stereotaxically injected an adeno-associated virus (AAV) that drives the genetically encoded fluorescent calcium indicator GCaMP6s in a Cre-dependent manner (AAV1.Syn.Flex.GCaMP6s). Injections were targeted to either the lateral or medial part of the Gi nucleus [Fig. 1A; coordinates: antero-posterior (A/P): 6.00 mm caudal to bregma, medio-lateral (M/L): 0.0 or 0.6 mm, dorso-ventral (D/V): -5.15 or 5.10 mm from the skull surface]. After injection, we implanted a gradient index (GRIN) lens above the injection site. The main concerns for endoscopic calcium imaging in the brainstem are minimization of tissue damage along the lens implantation path that could cause major impairments to the animal and reduction of brain motion related to the animal’s movements, which comes from the proximity to the chest of the recorded region. To handle these issues, GRIN lenses (0.6 mm or 1.0 mm diameter) were lowered very slowly (100 to 200 μm/min) after gently clearing the lens track with a blunt cannula at the same descending speed. To stabilize the tissue adjacent to the bottom of the lens, we equipped the lens tip with thin anchoring wires, which reduced movement artifacts in the imaging plane to a level that can be corrected post hoc (see Materials and Methods).

After implantation, animals recovered without motor impairment. The exact locations of the lenses and the transfected cells were verified histologically after the recordings (Fig. 1B and fig. S1). Both medial and lateral lens implantations (Fig. 1B, left and right, respectively) gave clear imaging fields with 2 to 12 detectable cells per field. Post hoc histology also indicated that GCaMP6s was not overexpressed, as judged from the absence of nuclear filling (Fig. 1B). After permanently fixing the appropriate docking system to the skull, a miniature microscope (Inscopix) was attached to the head of implanted animals to perform in vivo calcium imaging in freely moving conditions (Fig. 1, C and D). An example imaging field in Fig. 1D shows a contour map of the recorded cells with four clearly defined somata (all imaging fields can be seen in fig. S1). Calcium signals from single cells (e.g., Fig. 1D) were extracted using a standard signal demixing algorithm (see Materials and Methods) to compensate for contamination by out-of-focus fluorescence, which is unavoidable in one-photon imaging in thick tissue. However, similar calcium transient activity could be observed when directly extracting fluorescence for the regions of interest in which we could see a soma (fig. S2) and our analysis did not crucially depend on the demixing algorithm. Extracted signals could also be deconvolved (also shown in Fig. 1D) to give a more realistic time course of the firing rate variations that underlie the observed calcium transients (*32*–*34*). These surgical and recording methods allowed us to obtain the first monitoring of activity from V2a Gi neurons in seven mice.

### Dynamic subgroups of V2a Gi neurons are active at locomotor stops during treadmill running

**Fig. 2:**
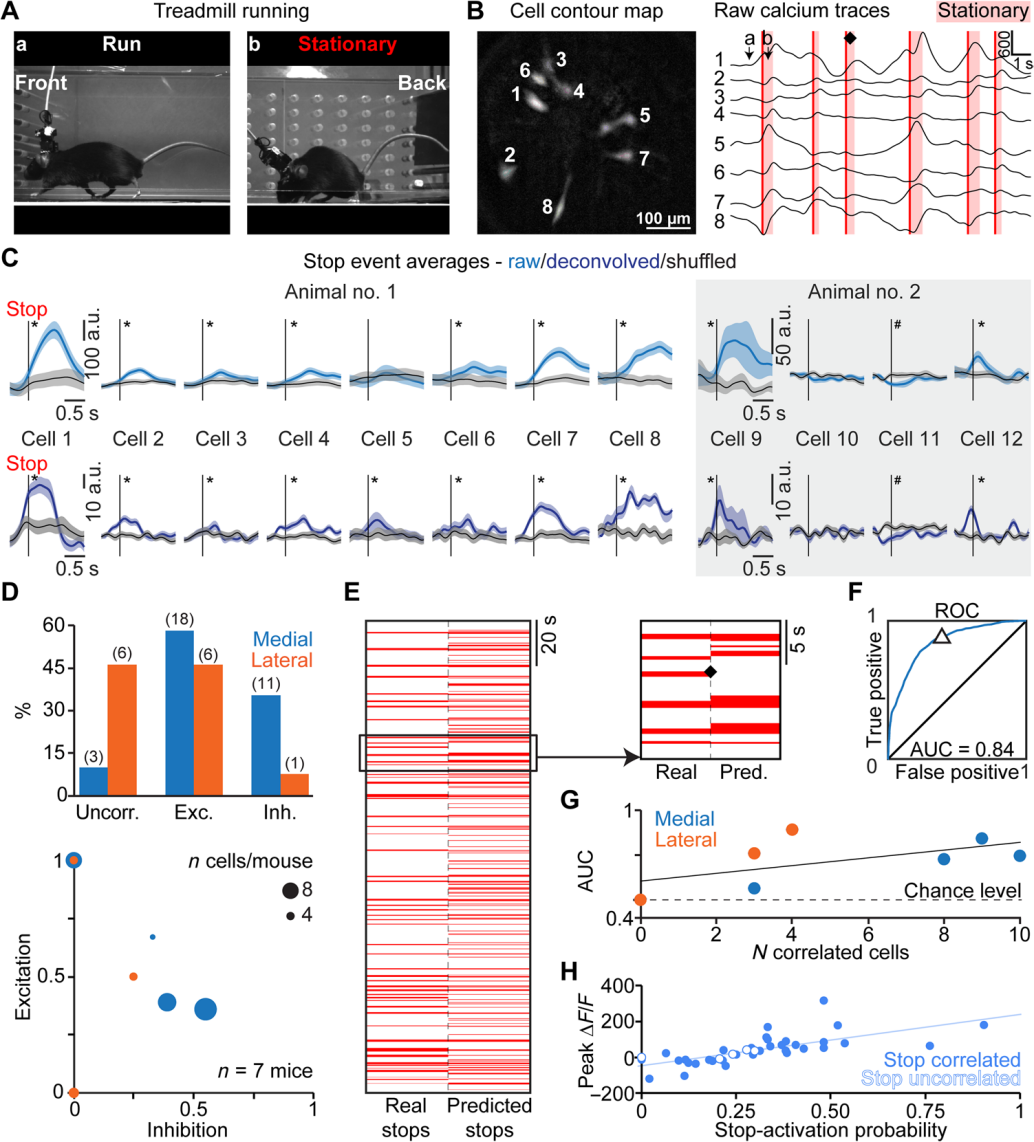
Activity of V2a Gi neurons correlates with spontaneous locomotor stops during treadmill running. Example mouse on treadmill (**A**) (photo credit: Joanna Schwenkgrub, CNRS). (**B**) Corresponding smoothed raw calcium traces (right) and cell contour map (left). Red vertical lines, stops; ♦, example stop event. (**C**) Associated average calcium transients at stop events are shown (left), together with another example animal (right); *, significant difference relative to time-shuffled data (gray shaded areas: SEM, *P* < 0.05). (**D**) Top: Fraction of V2a neurons uncorrelated, excited, or inhibited at stops. Distribution at lateral versus medial locations is significantly different (2 × 3 Fisher exact test, *P* = 0.0139). Bottom: Fraction of excited and inhibited cells per animal; dot size = number of recorded cells. (**E**) Real and predicted locomotion state (stationary = red bar). The magnification shows stops corresponding to traces in (B). ♦, unpredicted stop. (**F**) Receiver operating characteristic (ROC) for the same mouse. AUC, area under the curve. Δ = prediction performance in (E). (**G**) Number of cells per animal against performance of the stop classifier. Trend line in black. (**H**) Scatter plot of the peak Δ*F*/*F* (a.u.) for a stop versus stop-activation probability for all the visible cells (*n* = 39) recorded in six mice; resampling test, *P* < 0.05.

V2a neurons were previously implicated in the termination of locomotion when optogenetically activated (*15*), but their endogenous patterns of activity during naturally occurring halts have never been documented. To encourage animals to engage in episodic bouts of locomotion with minimal confounding behaviors, we first placed implanted mice on a motorized treadmill operating at 20 cm/s and animals were filmed from the side (Fig. 2, A and B). In this paradigm, animals exhibited long bouts of locomotion interleaved with short spontaneous periods of immobility, during which they translated backward on the treadmill (Fig. 2, A and B). Locomotion arrests were scored manually in the videos, blind to the calcium data, and cross-correlated with the activity traces of recorded cells (see Materials and Methods for details). This revealed synchronization of calcium transients with the onset of stop events for many recorded neurons (see example in Fig. 2B and in movie S1). However, stop-related activity was not systematic and individual neurons generally showed a clear calcium transient only for a fraction of stop events (Fig. 2B, see also heatmaps in figs. S3 and S4). Looking closer at neuronal activity averaged across stop events (Fig. 2C), about half of the imaged cells were significantly excited at stops, regardless of their medial or lateral position (Fig. 2D). The remaining cells were either inhibited or their activity was unchanged, with a larger fraction of inhibited cells located medially (34% of medial cells, *n* = 4 mice), while cells uncorrelated to stops were more often observed laterally (46% of lateral cells, *n* = 3 mice; Fig. 2D). Despite this heterogeneity, the activity of V2a neurons at the population level is informative about the locomotive state of the animal. To quantify this, we trained a support vector machine (SVM) classifier to predict whether the animal was walking or not based on the activity of all simultaneously recorded cells. The performance of the classifier was clearly above chance in more than two-thirds of the imaged animals (Fig. 2, E and F), as quantified by receiver operating characteristic (ROC) analysis (Fig. 2, F and G). Failure to predict some stop events suggests that a given subpopulation of activated cells only encodes a fraction of the stop events. About 90% of the cells excited at stops had an activation probability at any given stop event below 50% (Fig. 2, B and H). This shows that the V2a neuronal population encodes locomotor arrests in a heterogeneous and distributed manner, with many V2a cells being silent during single stop events.

### V2a Gi neurons are also active at locomotor arrests during spontaneous exploration

**Fig. 3:**
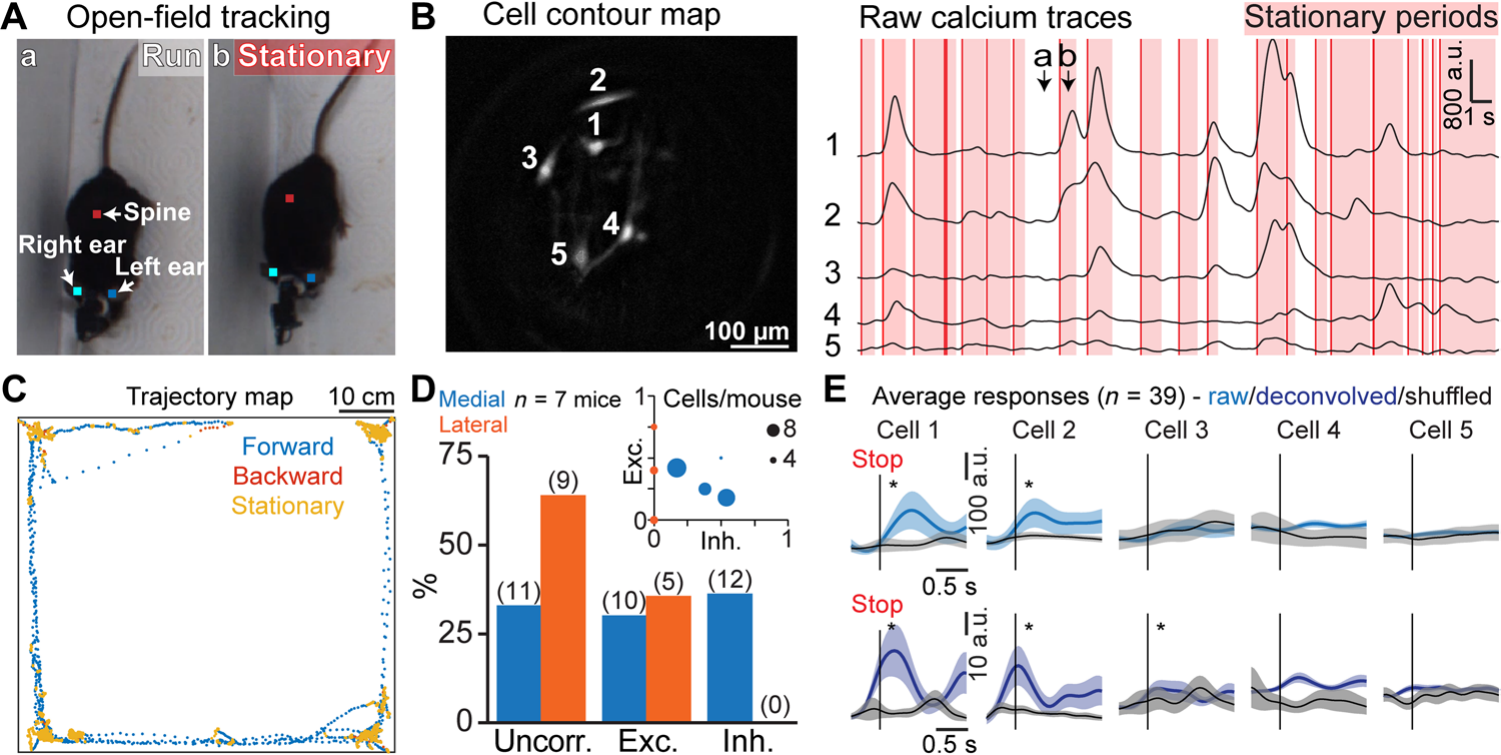
Activity of V2a neurons correlates with locomotor stops during open-field exploration. (**A**) Running (a) and stationary (b) postures of an example mouse (anatomical landmarks used for tracking are indicated with colored squares) in the open-field arena. Photo credit: Joanna Schwenkgrub, CNRS. (**B**) Example cell contour map (left) with corresponding raw calcium traces smoothed with Gaussian filter (half-width 150 ms) (right). Red vertical lines indicate stops; red shaded areas indicate stationary periods. (a) and (b) correspond to the behaviors shown in (A). (**C**) Trajectory map showing the position and locomotor state of an example animal during one experimental session; forward locomotion is in blue, backward locomotion is in red, and stationary state is in yellow. (**D**) Fraction of V2a neurons that are excited, inhibited, or not affected at stops for lateral (orange) or medial (blue) locations. The distribution of excited, inhibited, and uncorrelated cells was significantly different between medial and lateral populations (2 × 3 Fisher exact test, *P* = 0.017). Inset: Fraction of excited and inhibited cells per animal; the dot sizes correspond to the number of recorded cells. (**E**) Average calcium transients at stop events for all recorded cells from the example shown in (A) to (C) (raw and deconvolved data); * represents a significant difference relative to time-shuffled data (single shuffle averages in black, gray shaded areas represent SEM), *P* < 0.05.

We next addressed whether the activity of V2a neurons is also related to locomotor arrests in another behavioral context. For this, we imaged V2a neurons with the miniature microscope while the animals freely explored an open-field arena. Animals were filmed from above, and we tracked their position and locomotor speed using the DeepLabCut software [(*35*); see Materials and Methods for details; Fig. 3, A to C]. In this context, animals generally spend much more time stationary than in a locomoting state (Fig. 3, A and B), as previously reported (*15*). Nevertheless, many imaged cells had calcium transients synchronized with transitions from a walking state to a stationary state (Fig. 3B). As we observed in the treadmill running context, a large fraction of imaged V2a neurons were inhibited or their activity was unchanged at locomotor stops, with inhibited cells being more concentrated medially, and uncorrelated cells laterally (Fig. 3, D and E, and movie S2). Activity averaged across all stop events lasted for about 1 s (Fig. 3E), similar to the duration of the many short stops included in the analysis (minimal stop duration: 200 ms). However, restricting the analysis to longer locomotor arrests, of a minimal duration of 3 s, showed prolonged calcium transients, suggesting that stop-related V2a neurons remain active throughout the stationary state (fig. S5). Moreover, because locomotion and immobility bouts were more diverse in the open-field context, we explored whether other behavioral parameters (i.e., beyond the stop event itself) modulated the activity of V2a stop neurons. To do this, we correlated the amplitude of stop-related calcium signals with locomotion speed or deceleration before the arrest and found that the activity of some stop-modulated cells was weakly correlated with these parameters (maximum correlation coefficient, 0.3663; fig. S6). Also, a few cells were negatively correlated with the duration of locomotion before stop (fig. S6). The low positive (respectively negative) correlations observed mean that stop-related activity was higher (respectively lower) as the considered parameter increased, but that these influences were not strong enough to fully explain the variability observed in V2a neuron activity across stop events (see also single-cell examples in fig. S6). Last, we observed again that single V2a neurons were not active at every stop event (Fig. 3B), indicating that even during natural locomotion, V2a neurons behave as a heterogeneous, dynamic population.

### V2a Gi neurons are active at locomotor arrests with moderate global context dependency

**Fig. 4:**
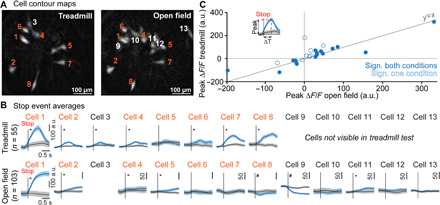
The correlation of V2a neuron activity with locomotor stops can be context independent. (**A**) Aligned cell contour maps of an example animal recorded in two behavioral contexts: treadmill running and open-field exploration. (**B**) Average calcium transients at stop events for all recorded cells from the example [raw data, cell numbering is the same as (A)] in both behavioral contexts; *n* corresponds to the number of detected stop events, and blank spaces indicate cells not detected in one of the conditions; * and ^#^ represent significant differences relative to time-shuffled data for excitation and inhibition, respectively (single shuffle averages in black, gray shaded areas represent SEM), *P* < 0.05. (**C**) Scatter plot of the peak Δ*F*/*F* for treadmill (ordinate) versus open-field (abscissa) stop-related calcium transients. Cells included in the plot are those visible in both and significantly correlated to stops in at least one of the behavioral contexts (*n* = 25 cells in six animals). Open circles denote cells with significant correlation to stops only in one behavioral context. Filled circles indicate neurons with significant correlation in both treadmill and open-field conditions. Inset: Definition of peak Δ*F*/*F* and time differences between locomotion arrest and the peak Δ*F/F* (Δ*T* stop).

The chronic implantation of a GRIN lens allowed us to follow the same neurons across imaging sessions (*36*). Taking advantage of this, we next addressed whether single V2a neurons are similarly active at locomotor stops in the two distinct behavioral contexts, treadmill running and open-field exploration (Fig. 4, A and B). We found that cross-session alignment of cells was not perfect, with only 27 cells visible in both contexts out of the 54 cells found in either context. Among these 27 cells, 25 exhibited activity that was significantly correlated with stops in at least one behavioral context. In this reduced population of 25 cells, we observed a strong correlation between peak activity at stop onsets on the treadmill and in the open field (Fig. 4C). Of these 25 cells, 8 cells were significantly inhibited in at least one context, and 6 of these were inhibited in both contexts. Likewise, of the 17 cells significantly excited in at least one context, 11 were significantly excited in both contexts. This indicates that despite the highly dynamic behavior of V2a neurons between individual stop events, the underlying function of each V2a neuron in locomotor control can be consistent over a long time scale and, at least for some cells, does not depend on the behavioral context in which locomotion arrest occurs.

### A subset of V2a neurons is active at locomotor start events

**Fig. 5:**
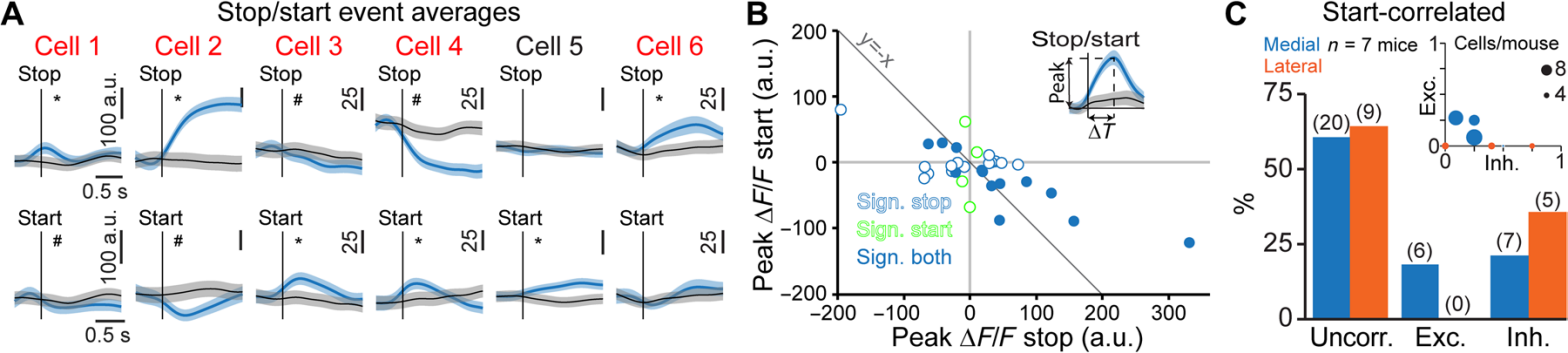
The activity of V2a neurons correlates with locomotor onsets during open-field exploration. (**A**) Average calcium transients at locomotor start and stop events from six example cells recorded in three different animals (raw data). Red lettering indicates stop-correlated cells; * and ^#^ represent significant differences relative to time-shuffled data for excitation and inhibition, respectively (single shuffle averages in black, gray shaded areas represent SEM), *P* < 0.05. (**B**) Scatter plot of the peak Δ*F*/*F* for start-related (ordinate) versus stop-related (abscissa) calcium transients in the open field. Cells included in the plot (*n* = 31 cells in six animals) are significantly correlated to at least one of the two behaviors, i.e., starts or stops of locomotion. Filled circles indicate neurons with significant correlation to both behaviors. Open circles denote cells significantly correlated only to one of them (start cells in green, stop cells in blue). Inset: Definition of peak Δ*F*/*F*. (**C**) Fraction of V2a neurons that are excited, inhibited, or uncorrelated at start events for lateral (orange) or medial (blue) locations. The absolute number of recorded cells is given in parentheses. Inset: Fraction of excited and inhibited cells per animal; the dot sizes correspond to the number of recorded cells. The distribution of excited, inhibited, and uncorrelated cells was not significantly different between medial and lateral populations (2 × 3 Fisher exact test, *P* = 0.24).

In the zebrafish, the homologous V2a brainstem neurons have been linked to locomotor initiation (*23*). While a similar function has not been demonstrated in mice, some V2a neurons are known to receive excitatory inputs from the mesencephalic locomotor region (*16*), a key neuronal center for engaging forward locomotion (*37*, *38*). It is therefore possible that the locomotor arrest evoked by bulk optogenetic activations dominates and obscures the function of numerically smaller V2a contingents. To investigate whether some V2a Gi neurons in mice are active at locomotor onsets, we averaged the activity of all imaged cells to the onsets of spontaneous locomotor episodes observed during open-field exploration. This analysis revealed that some V2a neurons are significantly excited or inhibited at locomotion onsets (Fig. 5A). In these examples, we noticed that cells excited at start events tended to be inhibited at stops (Fig. 5A). The opposite was also true, as cells excited at stops tended to be inhibited at starts (Fig. 5, A and B). Yet, opposing activity between starts and stops was not universal (e.g., cells 5 and 6 in Fig. 5A). Among 31 cells whose activity was significantly modulated at either starts or stops, 14 were significantly modulated for both behaviors (Fig. 5B), with opposite modulation signs for starts versus stops in all but one cell (Fig. 5B). As was the case with stops, cells activated at a start could also prolong their activity several seconds after the start, during continued locomotion (fig. S5), suggesting that they could contribute to the maintenance of the locomotion state. Also, a few cells showed a positive correlation of their activity with locomotion speed after starts (fig. S6E). We found that excitation at locomotor onset was less prevalent than excitation at locomotor offset in V2a neurons. Only 6 of 47 cells were significantly excited at starts against 15 of 47 excited at stops (2 × 3 Fisher exact test, *P* = 0.0716; Fig. 5C). Moreover, the magnitude of positive calcium transients at stops appeared larger than at starts (Fig. 5B). Together, this suggests that in the Gi, only a small fraction of V2a neurons is active at—and therefore possibly drives—locomotor initiation, possibly explaining why locomotor arrest is the dominating effect of bulk optogenetic activations.

### Some V2a Gi neurons are active during grooming

**Fig. 6:**
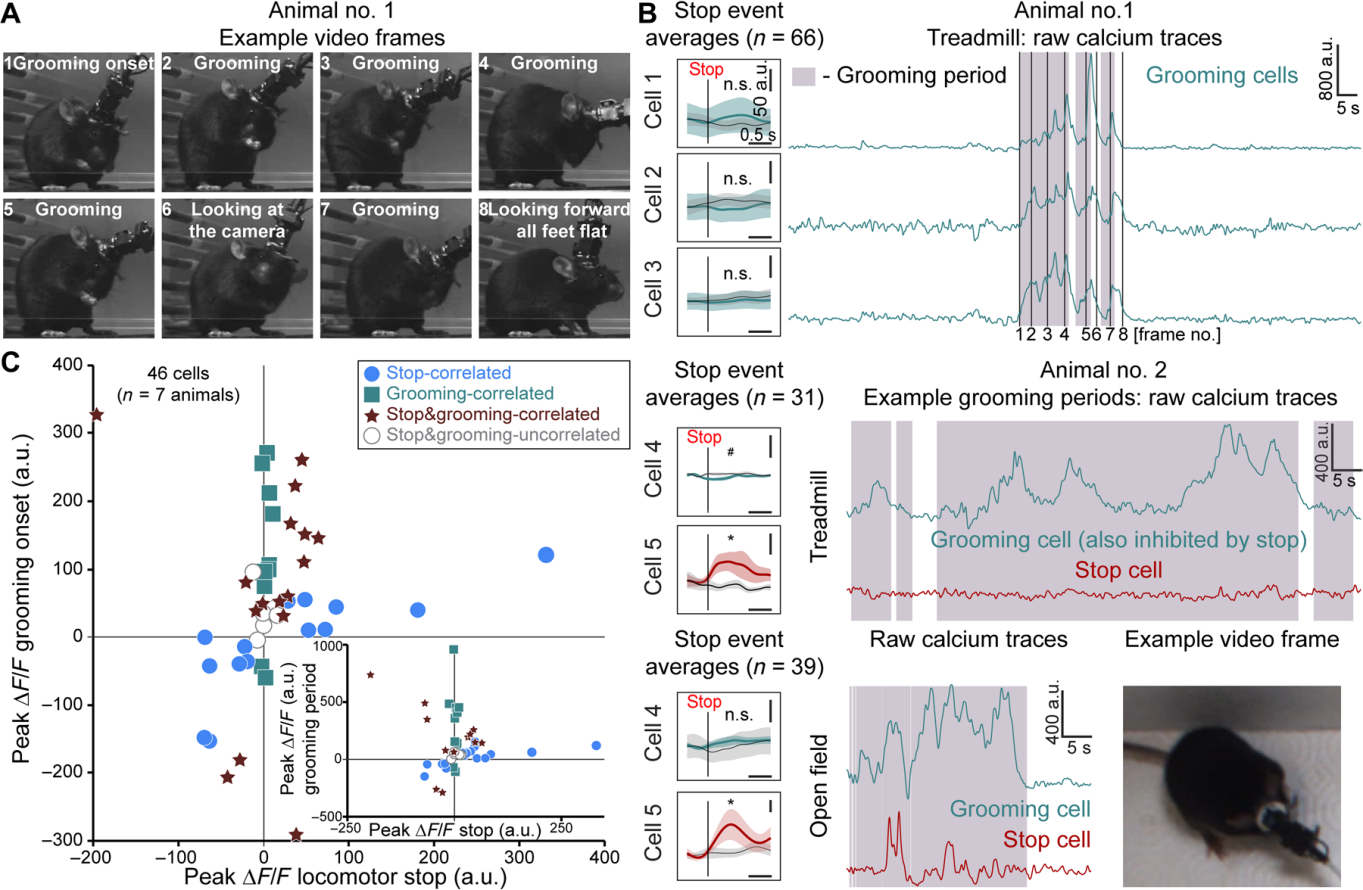
A fraction of V2a neurons are active during grooming. (**A**) Grooming postures (frame nos. 1 to 5 and 7) of example animal no. 1. (**B**) Top right panel: Calcium traces from three cells excited during grooming from the animal shown in (A). Frame numbers correspond to posture numbering from (A). Top left panel: Average of calcium transients at stop events of the same cells; n.s., nonsignificant correlation compared to time-shuffled data (*P* > 0.05). Bottom right panels: Calcium traces of the two example cells (grooming-correlated in turquoise, stop-correlated in red) recorded in animal no. 2 in treadmill (upper) and open-field (lower) conditions showing increased activity during grooming periods (shaded areas). Example grooming posture (lower right panel) from this animal. Bottom left panels: Stop event averages for example cells (raw data). * and ^#^ represent significant differences relative to time-shuffled data for excitation and inhibition, respectively (single shuffle averages in black, gray shaded areas represent SEM), *P* < 0.05. Photographs from (A) and (B) by Joanna Schwenkgrub, CNRS. (**C**) Scatter plot of the peak Δ*F*/*F* after grooming onset versus locomotor stops. The 46 cells included in the plot are all cells recorded in seven mice during free exploration (treadmill not engaged or open field). Inset: Scatter plot of the peak Δ*F*/*F* for a grooming period versus locomotor stops.

We reported above that a large fraction of V2a neurons have activity that does not correlate with either locomotor stops or starts. This prompted us to investigate whether some V2a neurons are associated with other behaviors. Inspection of the videos and their synchronized calcium traces suggested that the activity of some V2a neurons correlated with grooming. During the stationary periods that occurred on the treadmill apparatus when the treadmill was turned off after the running session, three of seven animals exhibited at least three epochs of grooming (annotated manually) defined by cleaning their limbs and/or fur, which can be easily observed when looking at the animal from the side. In an example mouse (animal 1; Fig. 6, A and B), movements and postures related to grooming (frame nos. 1 to 5 and 7) were synchronized with calcium transient onsets in four of five cells detected in the field of view (three examples in Fig. 6B, top right panel). For this animal, the activity of the same cells had no correlation with stops (Fig. 6B, top left panel), suggesting that some V2a neurons that are not linked with locomotion arrests may instead be mobilized during grooming. In a second animal, we identified in the same field of view one grooming cell that was inhibited during locomotor stops, together with a stop-activated cell that did not correlate with grooming (in turquoise and red, respectively, Fig. 6B, bottom panel). This indicates that locomotor-encoding V2a neurons and those related to grooming can be in close proximity. The same cells were also recorded in the open-field condition during which the relationship between their activity and grooming or stops was preserved (Fig. 6B, bottom lower panels). Four other mice did not groom during the short pauses of the treadmill experiment but showed grooming in the open-field context, which could also be scored manually. To quantify the activity of V2a neurons related to grooming and stops, we measured peak activity at grooming onset or during the whole grooming event and compared it to stop-related activity pooling together with data obtained from the open-field and treadmill experiment (in both experiments, grooming is observed during free exploration; 46 cells in seven mice, four mice in the open field and three on the treadmill). Twenty-five of 46 cells had significantly (assessed using resampling, see Materials and Methods) elevated activity at grooming onset, and 24 cells were still significantly activated 4 s later while grooming continued (Fig. 6C). Within those, cells with significant stop-related activity tended to have lower activity during grooming than cells without stop-related activity, most clearly when considering activity long after grooming onset (Fig. 6C, inset). Higher temporal resolution and analysis over a wider set of behaviors would be needed to determine the specific motor patterns that, during grooming, relate to V2a activity. Nevertheless, this observation reinforces the idea that V2a neurons include different functional subclasses, with some related to locomotor stops, some related to other motor behaviors, and possibly some encoding multiple motor actions. Thus, although bulk stimulation of V2a neurons mainly triggers locomotion arrests, this cell population may be involved in a much wider variety of motor programs.

## DISCUSSION

While optogenetic techniques for manipulating neuronal activity have greatly enhanced our ability to probe circuit function, imaging genetically identified cells within a neuronal ensemble remains essential for understanding the full complexity of the neuronal encoding of actions (*26*, *27*). In this pursuit, single-cell resolution optical recording methods in head-fixed animals have been widely used to study superficial brain structures, but it is challenging to apply these methods in deeper structures and even more difficult during unrestricted movement. Here, we perform the first functional imaging of genetically defined neurons in the ventral brainstem RF in behaving animals subjected to a locomotor task using cell type–specific expression of a calcium indicator combined with micro-endoscopy. This has the clear advantage of making cell identification straightforward while providing an indirect but reliable correlate of neuronal activity. We show that a large fraction of V2a neurons in the Gi nucleus is active at the onset of naturally occurring locomotor halts, corroborating earlier reports that used bulk optogenetic stimulations of these neurons to evoke locomotor arrests (*15*). Together, these findings firmly establish that a fraction of V2a Gi neurons are embedded in a circuit that is naturally mobilized during, and is causal to, locomotion arrests. This may be, at first glance, surprising for excitatory neurons because locomotor arrest is most commonly thought to rely on inhibitory descending neurons (*9*). Yet, a population of excitatory RS neurons whose activity correlates with, and is causal to, locomotor arrests has recently been characterized by electrophysiological recordings in the brainstem of the lamprey, a primitive representative of the vertebrate phylum easily amenable to cellular recordings (*39*, *40*). While the genetic identity of these stop-related RS neurons in the lamprey has yet to be determined, our results argue for a conserved active control of locomotor arrest by excitatory descending neurons across vertebrates. By directly observing the endogenous activity of populations of V2a neurons instead of only manipulating their activity, we were able to make a much more fine-grained dissection of their functional specificity than previous studies and this led to many unexpected findings. First, the population of V2a neurons modulated at each locomotor arrest varied across events (Figs. 2 and 3). Whether this owes to the limited sampling imposed by our small field of view or instead reflects that the mobilization of a few cells is sufficient to drive a motor response, as previously observed in other motor circuits (*41*, *42*), remains to be determined by examining larger neuronal ensembles. The variable activations of individual neurons could also reflect that several nonoverlapping V2a populations might be mobilized in distinct situations of behavioral arrest. A decrease or arrest in locomotor activity is associated with a variety of stimuli and behaviors, including defensive responses (*43*), meeting with obstacles (*9*, *44*), and sleep/wake cycle regulation (*45*, *46*). Mapping the structures that are upstream V2a Gi neurons using transsynaptic retrograde tracers (*47*) might provide complementary insights into the behavioral context(s) for their mobilization.

Another unexpected finding was that V2a neuron activity is sustained during stationary periods (fig. S5). Because genetic silencing of V2a Gi neurons was previously shown to lead to hyper-locomotive animals and to a reduced occurrence of spontaneous halts (*15*), V2a stop neurons may therefore also play a role in maintaining immobility. Furthermore, we found that a fraction of V2a Gi neurons exhibit activity that is correlated with locomotor starts, and cells activated at locomotor onset tend to be inhibited at locomotor arrest and vice versa. Within the scope of our behavioral analysis, one dominant population of V2a neurons (yet only ~50% of all cells on the treadmill and ~30% in the open field, overlapping with cells active during grooming) were those active at stops and inhibited at starts. One limitation of our study is the low temporal resolution of calcium signal recording, which is due to the slow rise and decay time constants of GCaMP6s and the 20-Hz frame rate of the video acquisitions. Decay kinetics can be compensated with deconvolution (*30*), but not rise kinetics. Deconvolved signals are thus smeared over at least 50 to 100 ms (i.e., about two frames). This is too imprecise to determine whether V2a neuron activity precedes or lags stop (or start) onset. However, simultaneous electrophysiological recordings of many genetically defined neurons that are intermingled with other cell types in the ventral brainstem are extremely challenging and have yet to be accomplished. Consequently, whether the activity of the start-related V2a neurons is causal to locomotor initiation, as reported in the zebrafish (*23*), or instead reflects locomotor-related inputs dedicated to sensory processes, remains to be investigated. This remark also applies to grooming-related V2a neurons, although the absence of photo-evoked movements resembling grooming in previous reports (*15*, *24*) may suggest noncausality. Locomotor stop neurons in the lamprey similarly show increased activity at locomotor onsets (*40*), but their direct activation only translates in a down-regulation of locomotion. Nevertheless, whether the V2a population hosts a numerically smaller subtype that may participate, together with other non-V2a neuronal classes (*5*, *6*, *48*, *49*), in locomotor initiation remains to be investigated. Furthermore, while V2a neurons may not be exclusively RS (*22*, *23*), our methodological approach cannot discriminate the activity of V2a neurons having different projection profiles. The advent of target-specific retrograde vectors should help in this endeavor (*50*), but larger fields of view will be needed to overcome the resulting sparsity of labeled neurons.

Our findings point toward an altogether more intricate model than one that just uses a transition-based stop-go encoding scheme. In this direction, we highlight that half of the imaged V2a neurons are not modulated at locomotor transitions. Although this could relate, as explained above, to their recruitment in situations of locomotor arrest not expressed in the presently examined behavioral contexts, we found that some of these cells were activated during grooming. Grooming and locomotion are mutually exclusive behaviors. It is therefore possible that V2a neurons not only impose locomotor immobility but also may allow animals to transit from ongoing locomotion to alternative motor actions that cannot be executed during walking and/or share similar motor effectors. Grooming, for instance, can only be performed while stationary and mobilizes stereotypical patterns of forelimb movements that are distinct from walking. It would be extremely informative to explore this further by correlating the activity of V2a neurons with other motor behaviors that require immobility and rely on descending circuits (i.e., orienting and reaching). This suggests that the current genetic handle on V2a neurons with a single transcription factor (Chx10) is not sufficient to disambiguate their functional diversity as well as their local or descending projection profile. Further examinations using projection-restricted manipulations, genetic screens, or single-cell RNA sequencing will undoubtedly reveal further diversity within this neuronal class, as recently achieved for other transcription factor–defined subgroups (*25*). In summary, our study demonstrates the feasibility of cell type–specific monitoring of neuronal activity in the complex RF and its great potential to advance our understanding of the neuronal dynamics underlying the orchestration of movements.

One shortcoming of our approach is that it cannot establish whether the stop-related activation is a unique property of V2a RS neurons, or if it is shared with other—i.e., non-V2a—neuronal classes. Because inhibitory RS neurons were previously shown to mediate behavioral arrest or muscular atonia (*9*, *46*), it is possible that their endogenous activity also correlates with locomotor arrests. However, we are not aware of any report having noticed a locomotor arrest from activating another, i.e., non-V2a, population of excitatory reticular neurons. Activating, in the Gi or adjacent nuclei, excitatory neuron collectivity without genetic specification was shown to either engage locomotion or evoke axial body movements (*6*). Therefore, V2a neurons, at least those located in the Gi nucleus, likely represent a divergent population of excitatory reticular neurons. Addressing the function and endogenous activity of non-V2a excitatory neurons with a dedicated intersectional viral strategy (*51*) will be needed to further dissect the functional specialization in the complex brainstem RF. Furthermore, our experiments are dedicated to V2a neurons located in the rostral medulla, i.e., in the Gi and adjacent nuclei. There, our viral strategy likely covers all Chx10-expressing neurons, but our one-photon imaging limits the detection to a few neurons within the field of view and/or at or near the focal plane. While we clearly did not image all V2a Gi neurons, the stop-related activity was persistent across animals, regardless of the medial or lateral implantation coordinates. Therefore, the reported sampling may be representative of the whole population. However, dedicated experiments will be needed to document the function of V2a neurons located more caudally and rostrally in the RF. Last, V2a neurons may also control other motor actions, including breathing (*22*) and orientation (*24*). Dedicated behavioral tests will be needed to examine whether these are also encoded by the V2a neurons that correlate with locomotor arrests, or instead by dedicated functional subgroups that possibly project to other motor circuits than the hindlimb controllers. Our result that grooming-related neurons were poorly correlated to locomotor arrests favors a model with motor action–specific subtypes of V2a neurons in the RF.

## MATERIALS AND METHODS

### Mice

Adult male *Chx10-Cre* mice (2 to 6 months old, 25 to 30 g), provided by S. Crone, K. Sharma, L. Zagoraiou, and T. M. Jessell [see (*15*, *30*, *31*)], were used in the study. All animals were group-housed in plastic breeding cages with free access to food and water, in controlled temperature conditions, and exposed to a conventional 12-hour light/dark cycle. All procedures in the experimental protocol were approved by the French Ethical Committee (authorizations 00275.01 and 2020-022410231878). Experiments were conducted in accordance with EU Directive 2010/63/EU. All efforts were made to reduce animal suffering and minimize the number of animals needed to obtain reliable results.

### Virus injection and GRIN lens implantation

All surgeries were performed with ketamine (80 mg/kg)–medetomidine (1 mg/kg) anesthesia. The animal’s body temperature was maintained at 36°C using a feedback-based thermal blanket with a rectal probe (Rodent Warmer X1, Stoelting). *Chx10-Cre* animals were placed in a stereotaxic frame (Stoelting), and two injections of 300 nl of AAV1.Syn.Flex.GCaMP6s (titer: 1.937 × 10^12^ to 2.906 × 10^12^ Genome Copies/ml; Vector Core) were made at 50 nl/min at two locations vertically separated by 500 μm. GCaMP6s was chosen over GCaMP6f for its higher sensitivity of spike detection (*52*), while its longer decay kinetics can be compensated with deconvolution, yielding better temporal precision than raw GCaMP6f signals. We used glass micropipettes and a programmable pump (Micro 4; World Precision Instruments) to deliver the virus into the Gigantocellularis (Gi) nucleus of the ponto-medullary RF. Stereotaxic coordinates used to target laterally/medially located V2a Gi neurons were as follows: A/P, −6.0 mm; M/L, 0.6 mm/0 mm; D/V, 5.0 mm and 5.5 mm. One week after the viral injection, a GRIN lens (0.6 mm by 7.3 mm or 1.0 mm by 9.0 mm; Inscopix, Palo Alto, CA) was implanted above the injected site to enable optical access to changes of fluorescence (Δ*F*/*F*) of individual V2a neurons by epifluorescence micro-endoscopy. This procedure comprised a series of preparatory steps. First, space for the lens in the brain was made by lowering a blunt needle of a size corresponding to the lens at 100 to 200 μm/min (tissue located 300 μm above the imaging site remained untouched). Second, four short tetrodes (Sandvik Precision Fine Tetrode Wire) were attached to the end of the lens (protruding by 500 μm) with optical glue (Norland Optical Adhesive 68, Thorlabs) to reduce motion artifacts and stabilize the brain tissue during recording [approach developed by J. Courtin (Luthi laboratory, FMI, Basel, Switzerland) and adapted by S. Gulati (Inscopix R&D, Palo Alto, USA) to GRIN lens]. Last, the inserted lens was sealed to the skull with dental cement (Super-Bond C&B, Sun Medical Co. Ltd.) and a low-viscosity composite (Flow-It ALC, Pentron), and its surface was protected with a polymerase chain reaction (PCR) tube cap (Eppendorf). A head post, allowing transient head fixation of the animal to facilitate base plate positioning and miniature microscope mounting, was also attached to the skull with Super Glue-3 (Henkel) and dental cement. After lens implantation, animals were singly housed to prevent damage to the implant. Some animals displayed behavioral abnormalities for 24 to 72 hours after lens implantation surgery, including reduced mobility and circling behavior. In all cases, these abnormal behaviors were no longer present after the recovery period, during which we carefully followed the animals and provided diet gel and analgesia. Four to 6 weeks following surgery (depending on the clarity of the field of view), the baseplate (Inscopix) for the nVista microscope (v2.0, Inscopix) was mounted onto the animal’s head, ensuring the best imaging field of view. After behavioral experiments (treadmill running and open-field exploration), mice were anesthetized with Euthasol (150 mg of pentobarbital/kg) and perfused with 4% paraformaldehyde (PFA) in phosphate-buffered saline (PBS). Whole heads were postfixed overnight in 4% PFA at 4°C. Brains were then dissected and transferred to PBS. Coronal sections (100 μm thick) were obtained with a vibratome (Leica) and underwent Nissl staining (NeuroTrace 640, Invitrogen; applied at 1:500 for 4 hours). Confirmation of both lens positioning and GCaMP6s expression was obtained using a Leica SP8 laser-scanning confocal microscope (Fig. 1B and fig. S1). Overall, our success rate for this surgery, including method development, was 23%: 7 imaged mice for 31 implanted. Most common failures were (i) the lack of active cells in the field of view due to lens or baseplate mislocalization or overexpression of GCaMP6s before the appropriate dilution of the virus was found, (ii) scratched/chopped GRIN lens due to the animal removing the protection cap, (iii) lost implant before baseplate implantation, and (iv) excessive movement artifacts in the imaging plane in test experiments, especially when the tip of the lens was not equipped with thin anchoring wires.

### Behavior and imaging

Animals were habituated to the nVista miniature microscope attached to their head before experimental sessions. GCaMP6s was excited with a light-emitting diode (LED) providing blue light at 475 nm (±7 nm, one-photon excitation), and fluorescent light was collected through a 535 ± 25–nm emission filter and a complementary metal-oxide semiconductor (CMOS) camera chip, all embedded in the miniature microscope. Following 10 min of free exploration of the environment (treadmill not engaged, or open field) and 3-min light habituation with LED on, recordings of neuronal activity were acquired at 20 images per second with a 50-ms exposure time, for 5 to 13 min depending on animal endurance and/or motivation (there were no training sessions before). Mouse behavior was filmed from the side using a CMOS camera (Jai GO-5000C-USB) at 20 or 50 images per second, and images were streamed to disk on a computer using the 2ndLook software (IO Industries). The acquisition start of behavioral video recordings was hardware-triggered by the start of the nVista calcium recording. We used a custom-made motorized treadmill (Scop Pro, France) with a belt of 6-cm width by 30-cm length, set at a speed of 20 cm/s. The animals were allowed to recover (treadmill off) during recordings, but only data acquired from a treadmill-engaged (treadmill on) period were further used for stop analysis. Neuronal activity within the breaks was assessed only for animals (*n* = 3) that exhibited at least three epochs of grooming behavior (Fig. 6). For open-field conditions, animals were left to explore freely in a customized open-field arena (size, 62 cm × 68 cm) while being filmed from above at 20 or 50 frames per second.

### Behavior and calcium imaging analyses

#### Stop definition

Locomotion arrests (stops) during treadmill running were scored manually. The first frame with all limbs flat-footed was considered as the onset of a locomotor stop, whereas the initiation of a hindlimb movement was considered as the onset of locomotor initiation (or start). These assignments were carried out blind to the calcium recordings. To define stops in the open field, we used DeepLabCut (*35*) and a custom MATLAB script. The oriented speed of each animal in the arena was calculated on the basis of the tracking of the position of the ears and spine (Fig. 3A). Briefly, the vector defined by the spine and the point in between the ears was the body orientation, and the mean speed in this direction was computed across time. Then, we set a speed threshold at ~3 cm/s (2.8 to 3.2 cm/s, depending on slight changes in the field of view of the camera) to define forward locomotion (>3 cm/s), backward locomotion (<−3 cm/s), and immobility. Stops were identified as onsets of immobility lasting at least 200 ms (3 s for long stop events) following a minimum run duration of 250 ms (100 ms for long stop events), during which average speed had to be above 5 cm/s (selection of suprathreshold speed variations). The latter condition was not used for long stop analysis to increase the number of detected events.

#### Start definition

If not mentioned otherwise, we identified transition from a stationary state to forward locomotion (starts) in the open field following the same protocol as for stop definition. Analysis was based on the exact same behavioral video recordings while using a custom MATLAB script. The parameters used for start event averages calculation were as follows: 250 ms, minimal duration animal spent at stationary state; 200 ms, minimal duration of locomotion period; a speed threshold defining the mean speed of the mouse during stationary condition before start of locomotion was established as lower than ~2 cm/s (1.9 to 2.1 cm/s).

#### Calcium imaging analysis

Extraction of calcium signals from individual cells was performed with the Inscopix Data Processing software by applying the following steps. First, the raw movies were spatially down-sampled (×2) and filtered with a spatial band-pass filter (low cutoff: 0.005; high cutoff: 0.500 pixel^−1^), removing low and high spatial frequency content from microscope movies. Then, a rigid image registration algorithm was applied to spatially realign successive frames based on the high contrast region of the image (blood vessels) and thereby correct for residual horizontal movement artifacts. After this step, fluorescence signals from each pixel were normalized by its time-averaged mean (Δ*F*/*F* calculation). Last, signals from individual cells were identified using the standard principal components analysis (PCA)/independent components analysis (ICA) independent signal demixing algorithm (*36*), after which independent signals corresponding to neurons were manually selected on the basis of the locality of source pixels and the presence of clean, asymmetric calcium transients in the resulting traces. Note that the scaling of PCA/ICA traces is different from Δ*F*/*F* signals, as it depends on the loading of each independent component. Corresponding values are thus given in arbitrary units. Recordings with no visible cells or motion-induced artifacts that persisted after the correction were excluded (fig. S3). The final dataset from one behavioral condition per animal was from one recording session. Raw calcium data were preprocessed using a Gaussian filter (MATLAB) with a 150-ms half-width. We also deconvolved the calcium signal to show an estimate of the time course of the firing rate using the equation *S*_d_ *=* τ*dS/dt + S* (*32*–*34*), where *S*_d_ is the deconvolved signal, *S* is the raw signal, and τ = 0.5 s is our estimate of GCaMP6s decay time in V2a neurons, directly measured on traces obtained after PCA/ICA. GCaMP6s transients have a long exponential decay, whose time constant depends on cell type. The transients also have a nonnegligible rise time typically between 50 and 100 ms (*52*). Deconvolution estimates the time course of the firing rate signal after canceling the slow decay kinetics. However, deconvolution does not cancel the slow rise time kinetics. Therefore, the deconvolved signal is an estimate of the cell firing rate filtered with a 50- to 100-ms exponential kernel. This means that deconvolved firing rate estimates lag by 50 to 100 ms (up to two imaging frames) the actual firing rate of the neuron.

#### Stop prediction

A linear SVM classifier was trained to predict locomotor stops based on population activity vectors obtained from the neuronal activity recorded in single animals during stop events. Cross-validation of the predictions was done by a jack-knife method in which the calcium data obtained from single animals were divided into training (80%) and testing (20%) chunks. Training chunks were specified randomly. The jack-knife was carried out five times and averaged to obtain the final performance results.

### Statistical analysis

Nonparametric tests (resampling test, Fisher exact test, and Wilcoxon rank-sum test) described in figure legends and in Results were performed in MATLAB (MathWorks). The Fisher exact test was with online code from Cardillo G. (2007) available in the MATLAB File Exchange platform (www.mathworks.com/matlabcentral/fileexchange/15399). All tests are two-sided. The results were considered statistically significant for *P* values less than 0.05 (*P* < 0.05). A resampling test was used to evaluate the significance of the single cell activity at stops (in both treadmill and open-field conditions), starts (in open-field condition), and grooming (in both treadmill off periods and open-field conditions). In this procedure used for stop analysis, first the minimal and maximal ∆*F*/*F* values of stop event averages (for treadmill: only during treadmill on periods; for open field: full recording) were computed within a 1.5-s time window, which begins 0.25 s before the defined stop. Then, we randomly shuffled the stop times with respect to the calcium data 1000 times within a specific behavioral recording, respecting the time periods in which we analyzed stops in the particular behavioral conditions (treadmill or open field). Last, we compared min and max ∆*F*/*F* of the actual stop event to the shuffled distributions. A maximum or minimum Δ*F*/*F* outside of 95% of the shuffles was considered a significant reponse. Stop event averages are presented as a mean value ± SEM. The same analysis was performed on open-field endoscopic data regarding start event averages. We also used the resampling test to evaluate the significance of the single-cell correlations to grooming during treadmill off periods and open-field conditions. The maximal ∆*F*/*F* values of event averages during grooming periods were computed during an extended time window (4.25 s), whereas the analysis of peak ∆*F*/*F* at grooming onset was performed with a 1.5-s time window (the same time window as for stop event averages). We also calculated stop-activation probability of all visible cells recorded in six animals (one animal with no stop-correlated cells was discarded from this analysis) during treadmill running (see Fig. 2H). A coded stop was defined by peak ∆*F*/*F* bigger than five SDs of the baseline ∆*F*/*F*. The total number of coded stops was then divided by the total number of stops for each cell to obtain the stop-activation probability.

## Supplementary Material

http://advances.sciencemag.org/cgi/content/full/6/49/eabc6309/DC1

Movie S1

Movie S2

Adobe PDF - abc6309_SM.pdf

Deep imaging in the brainstem reveals functional heterogeneity in V2a neurons controlling locomotion

## SUPPLEMENTARY MATERIALS

Supplementary material for this article is available at http://advances.sciencemag.org/cgi/content/full/6/49/eabc6309/DC1

## Appendix

View/request a protocol for this paper from *Bio-protocol*.
