# Supplementary material for: Deep imaging in the brainstem reveals functional heterogeneity in V2a neurons controlling locomotion
Source: Sci Adv. 2020 Dec 4;6(49):eabc6309. doi: 10.1126/sciadv.abc6309 (PMC7821901; doi:10.1126/sciadv.abc6309)
Supplement: http://advances.sciencemag.org/cgi/content/full/6/49/eabc6309/DC1 [file supp_6_49_eabc6309__index.html]

Science Advances | Science AdvancesAAASSearchScience AdvancesMenu

## Supplementary Materials

# Deep imaging in the brainstem reveals functional heterogeneity in V2a neurons controlling locomotion

Joanna Schwenkgrub, Evan R. Harrell, Brice Bathellier, Julien Bouvier

Download Supplement

**The PDF file includes:**

- Figs. S1 to S6
- Legends for movies S1 and S2

**Other Supplementary Material for this manuscript includes the following:**

- Movie S1
- Movie S2

**Files in this Data Supplement:**

- Adobe PDF - abc6309\_SM.pdf
