# Supplementary material for: Deep imaging in the brainstem reveals functional heterogeneity in V2a neurons controlling locomotion
Source: Sci Adv. 2020 Dec 4;6(49):eabc6309. doi: 10.1126/sciadv.abc6309 (PMC7821901; doi:10.1126/sciadv.abc6309)
Supplement: http://advances.sciencemag.org/cgi/content/full/6/49/eabc6309/DC1 [file supp_6_49_eabc6309__1.pdf]

Science Advances
AAAS

advances.sciencemag.org/cgi/content/full/6/49/eabc6309/DC1

Supplementary Materials for

Deep imaging in the brainstem reveals functional heterogeneity in V2a neurons controlling locomotion

Joanna Schwenkgrub, Evan R. Harrell, Brice Bathellier*, Julien Bouvier*

*Corresponding author: Email: brice.bathellier@cnrs.fr (B.B.); julien.bouvier@cnrs.fr (J.B.)

Published 4 December 2020, *Sci. Adv.* **6**, eabc6309 (2020)
DOI: 10.1126/sciadv.abc6309

The PDF file includes:

Figs. S1 to S6
Legends for movies S1 and S2

Other Supplementary Material for this manuscript includes the following:

(available at advances.sciencemag.org/cgi/content/full/6/49/eabc6309/DC1)

Movies S1 and S2

Supplementary Materials

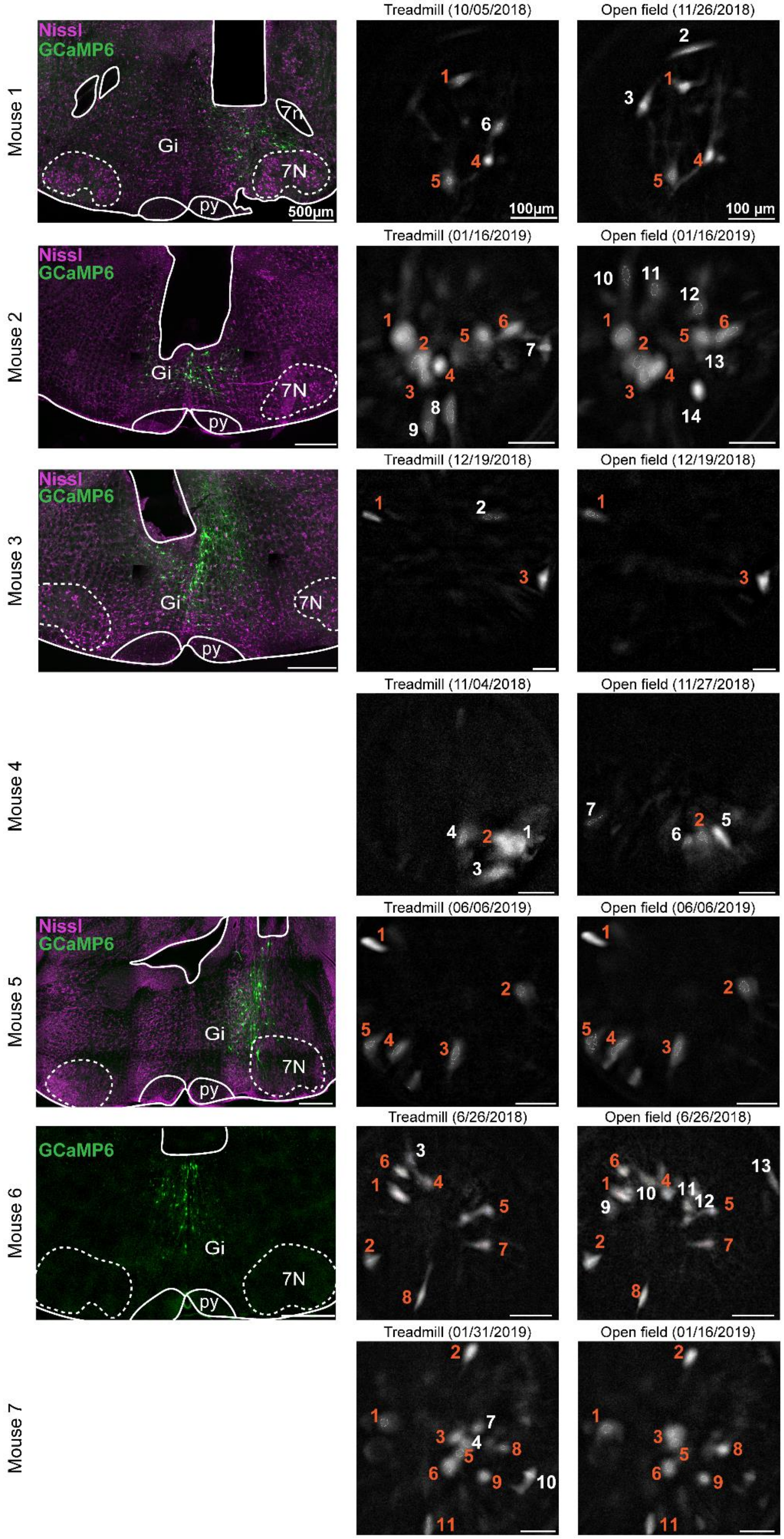

Fig. S1. Histology of animals used in the study and stability of recorded fields of view across experiments.

Histological data (coronal sections, 100 µm; unavailable for mouse no. 5 and 7) with the corresponding cell contour maps of all animals used in the study to the right. For each recorded mouse, the map of cell contours extracted by PCA/ICA for the recording sessions on the treadmill (left) and in the open field (right) are displayed. The two sessions were done on the same day or on different days depending on the animal (dates are given above the images). Cells that were seen in both sessions are numbered with red digits and cells that were seen in only one session are numbered with white digits.

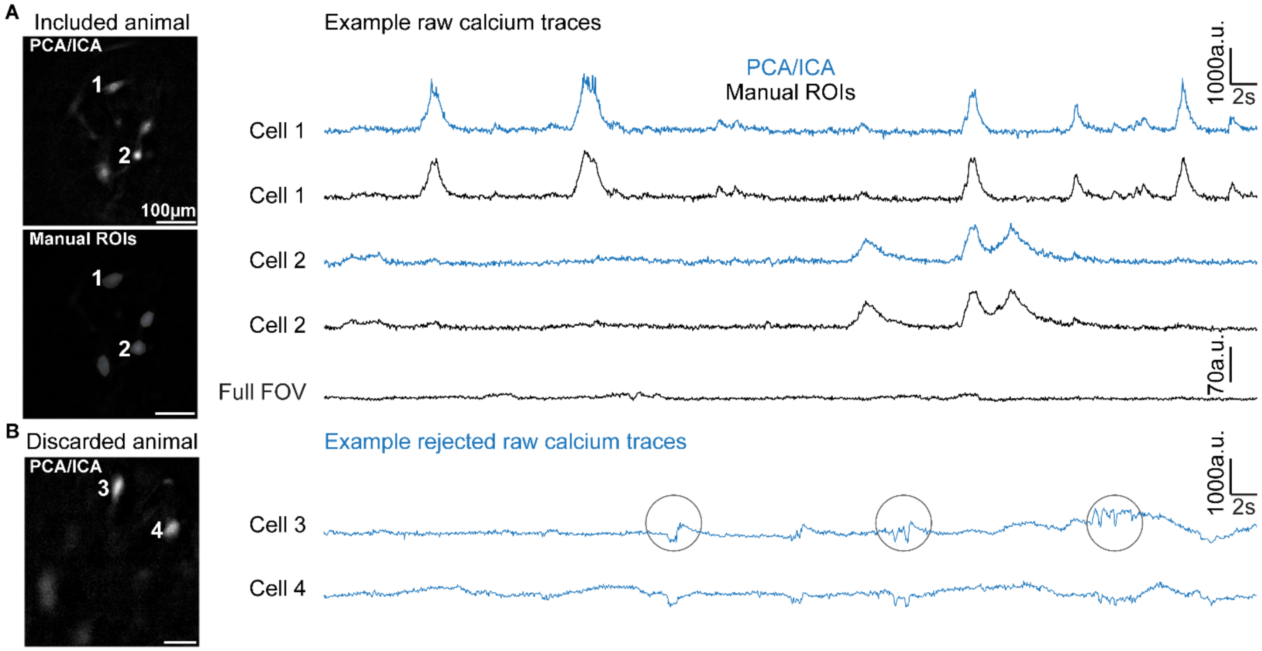

Fig. S2. Example traces for stable and motion-contaminated recordings.

(**A**). (top left) Example cell contour map obtained after PCA/ICA demixing. (bottom left) Same image overlaid with the 4 masks of manually selected regions-of-interest. (right) Raw calcium traces derived from 2 manually selected regions of interest (black) are compared to PCA/ICA traces (blue) for regions labelled as 1 and 2 on the contour map. In all traces, motion correction was applied but it had little impact given the stability of the recording. Full FOV: time course of the signal when averaged over the entire field of view. (**B)** Examples of PCA/ICA traces after motion correction from 2 regions of interest selected from an animal in which persistent motion artefacts (indicated by black circles) clearly contaminated the signal. This animal and these regions of interest were therefore not included in our analysis.

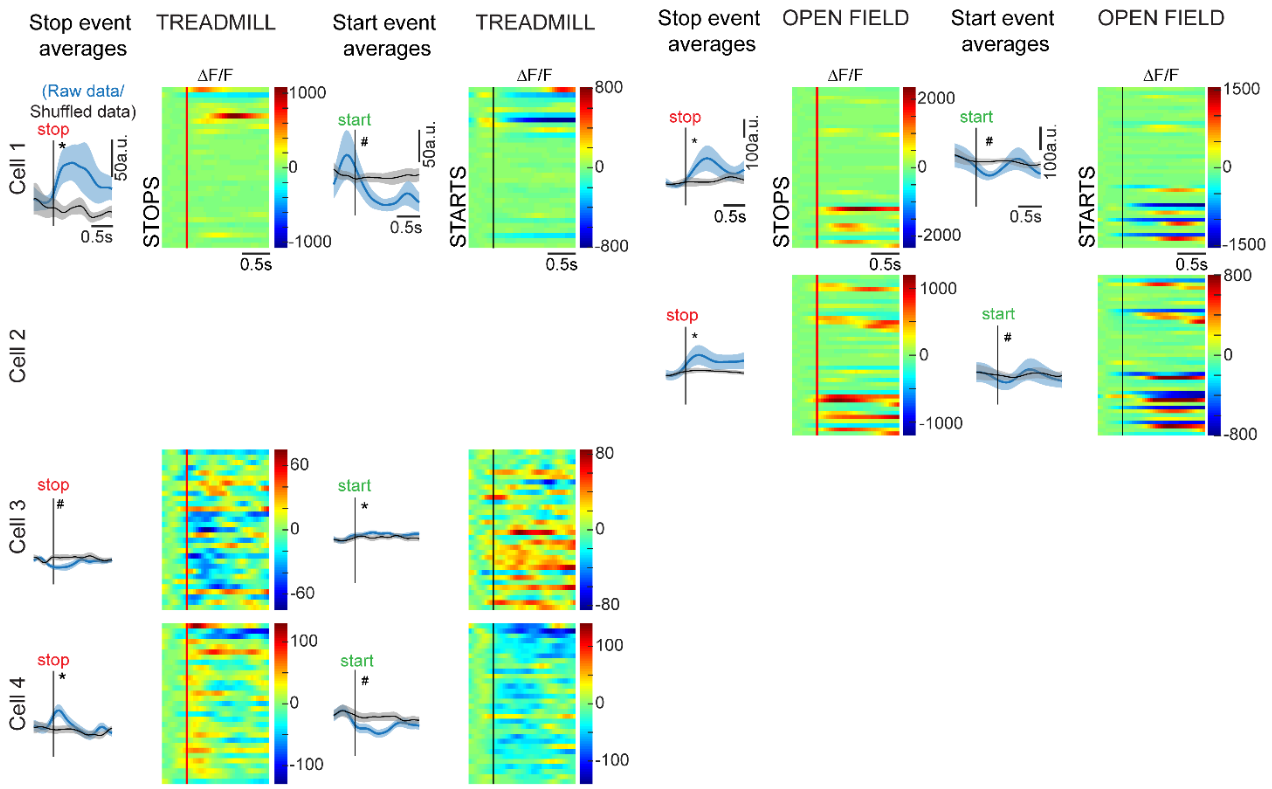

Fig. S3. Variability of stop- and start-related activities across single trials for sample neurons. ΔF/F heatmaps for stop-correlated cells of the animal presented in **Fig. 3**. Each row corresponds to a single trial. Data from the treadmill (left) and open field (right) contexts are displayed. On the heatmaps, vertical red lines indicate the timing of stop events and vertical black lines indicate the onset of locomotor start events. Averaged activity traces displayed to the left of ΔF/F heatmaps for stop-correlated cells in open field context are reproductions of **Fig. 3**. Calcium traces for all events were normalized to the average ΔF/F values across 5 frames beginning 1 second before the event (stop or start events).

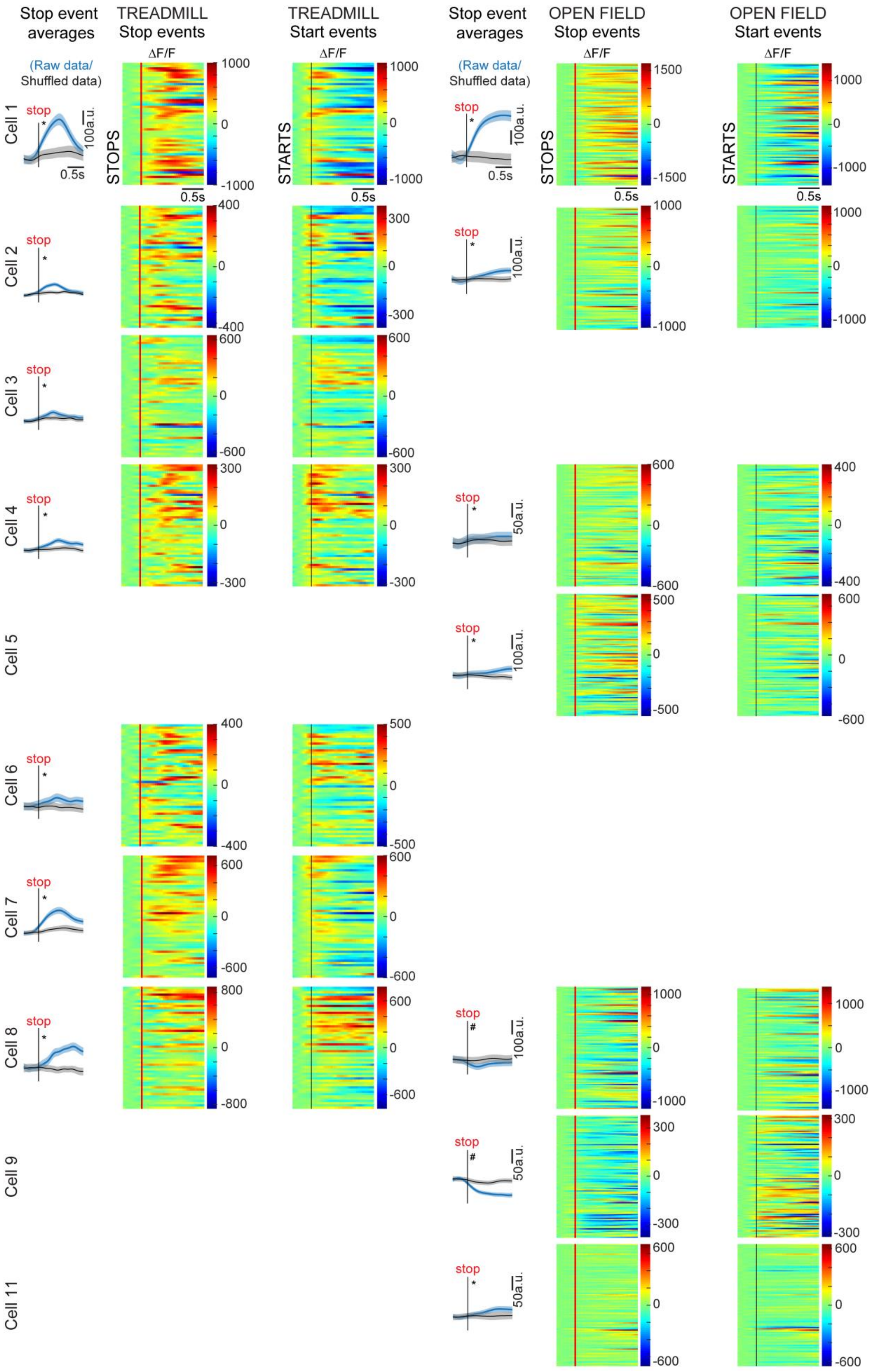

Stop event averages
TREADMILL Stop events
TREADMILL Start events
Stop event averages
OPEN FIELD Stop events
OPEN FIELD Start events
(Raw data/ Shuffled data)
ΔF/F
STOPS
STARTS
stop
100a.u.
50a.u.
0.5s
Cell 1
Cell 2
Cell 3
Cell 4
Cell 5
Cell 6
Cell 7
Cell 8
Cell 9
Cell 11

Fig. S4. **Variability of stop- and start-related activity across single trials for further sample neurons.**

ΔF/F heatmaps for stop-correlated cells presented in **Fig. 4**. Each row corresponds to a single trial. Data from the treadmill (left) and open field (right) contexts are displayed. On the heatmaps, vertical red lines indicate the timing of stop events and vertical black lines indicate the onset of locomotor start events. Averaged activity traces displayed on the left are reproductions of **Fig. 4**. Calcium traces for all events were normalized to the average ΔF/F values across 5 frames beginning 1 second before the event (stop or start events).

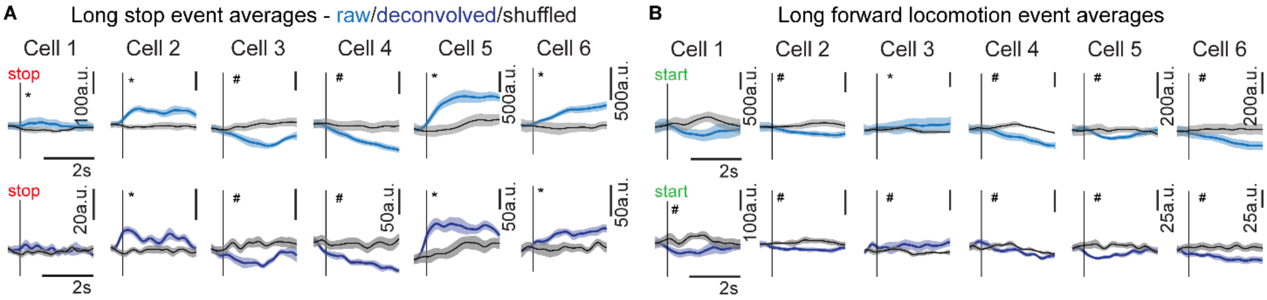

Fig. S5. Stop- and start-related V2a neurons remain active long after event onset.

(**A)** Average of calcium transients during long stop events from six example cells recorded in 4 different animals in the open field (raw and deconvolved data) for a minimal stop duration of 3s; * and # represent significant differences relative to time-shuffled data for excitation and inhibition respectively (single shuffle averages in black, grey shaded areas represent SEM), p<0.05.

(**B**) Average of calcium transients during long locomotor bouts (minimal forward locomotion duration of 3s) after a start event in six cells recorded in 3 different animals during open field exploration (raw and deconvolved data). Among 42 cells recorded in 6 animals, only these 6 cells were significantly active. The number of detected events varied between 5 and 19 per animal; * and # represent significant differences relative to time-shuffled data for excitation and inhibition respectively (single shuffle averages in black, grey shaded areas represent SEM), p<0.05.

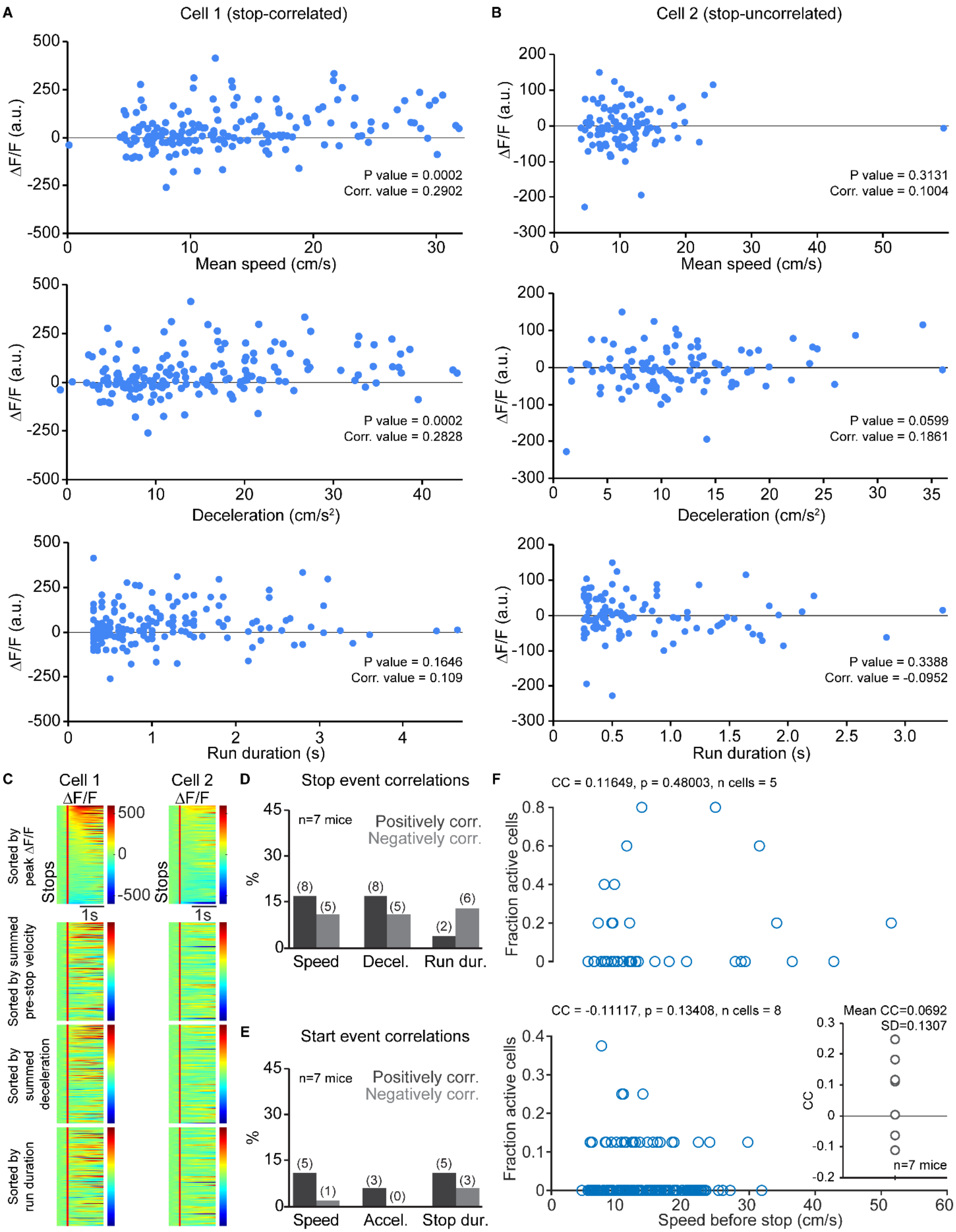

Fig. S6. Correlations between activity of V2a neurons and locomotion parameters.

(**A**) Plot of ΔF/F value at locomotor stop events (mean between 0 and 0.5 s after stop onset) against mean speed (top) or deceleration (middle, mean during the 250 ms that precede stop onset) before

stop or against duration of the locomotor bout before stop (bottom) for a sample cell showing positive correlation with speed and deceleration. Correlation value and associated p-value (significance assessed by correlation distribution observed with the same number of observations of a random variable, Matlab) are displayed on the graphs. Data is derived from open field sessions. (**B**) Same as A. but for a non-correlated cell. (**C**) Heatmaps of single trial activities for the cells shown in A (left) and in B (right). Trials are sorted according to ΔF/F after stop (top), speed (2nd row), deceleration (3rd row), and duration of locomotion before stop (run dur.). (**D**) Fraction of the 47 cells recorded in the open field context (7 mice) that show positive (black) or negative (grey) correlation with pre-stop speed and deceleration or with run duration. (**E**) Same as D but for start events (mean ΔF/F calculated between 0 and 0.5 s after start onset; mean speed and acceleration calculated between 0 and 250 ms after start onset). (**F**) Plot of locomotion speed against the number of significantly activated cells (ΔF/F > 1.5 baseline SD) in each trial for two sample mice. All correlation values are given in the inset, with mean and standard deviation (SD). No significant correlation was observed between speed and ΔF/F after stop.

Movie S1. Example mouse during treadmill running together with calcium traces of the simultaneously imaged cells.

Example Chx10-Cre mouse during treadmill running (top panel) with representative ΔF/F movie (bottom left) showing V2a neuron activity with corresponding calcium transients from 4 GCaMP6s-expressing cells (bottom right; cells 1 and 3 indicated in the ΔF/F movie). The stationary periods (red square in behavioral recording) and stops (red vertical lines in calcium data) are indicated. The video is played at half of the original speed.

Movie S2. Example mouse during open field exploration together with calcium traces of the simultaneously imaged cells.

Example Chx10-Cre mouse during open field exploration (top panel) with representative ΔF/F movie (bottom left) showing V2a neuron activity with corresponding calcium transients from 5 GCaMP6s-expressing cells (bottom right; cells 1-4 indicated in the ΔF/F movie). The stationary periods (red square in behavioral recording) and stops (red vertical lines in calcium data) are indicated. The video is played at half of the original speed.
